# Fungus of the Brain

**Published:** 1844-01-13

**Authors:** 


### FUNGUS OF THE BRAIN.

At the Sheffield Medical Society, October 19th, 1843, Dr. Favell exhibited a specimen of fungous tumour of the brain. The subject of the disease was a sober, temperate man, aged forty, who had for several years suffered from epilepsy and occasional attacks of meningitis. About seven years ago the left eye became affected with strabismus, and for a short time before his death the whole of the left side of the body was paralytic. His chief complaints were severe headache and vertigo, and these symptoms were from time to time so much mitigated, as to enable him occasionally to follow his employment as a joiner. During his illness his wife had two confinements. The children were weakly, and both died a few months after birth. He never suffered from lowness of spirits; the intellect was not impaired, but his temper was particularly irritable and hasty.

The tumour, which presented the usual characters of medullary sarcoma, occupied a space of three inches in length, and about an inch and a half in breadth, along the outer surface of the right hemisphere, and occasioned a corresponding depression in the substance of the brain. It was very firmly attached to the inner surface of the dura mater, and also to the superior surface of the arachnoid. The dura mater, in the neighborhood of the tumour, was intensely injected, as was also the arachnoid. The latter membrane was likewisely greatly thickened. The substance of the brain on the right side, especially in the situation of the fungus, was considerably softened. The rest of the brain was heatlhy.

### GRINDER'S LUNGS.

Dr. Favell exhibited—

1. A portion of the superior lobe of the left lung of a razor-grinder, who had died from pneumonia of the right lung, without having previously suffered from the usual symptoms of grinders' asthma. The portion shown was of a dark grey colour, and exceedingly dense, having the consistence of India-rubber. The under surface of the incised lung (where it was cut off from the rest) exhibited numerous small, dark-coloured bodies, like granulations.

2. A portion of lung from the body of a table knife-grinder. This specimen was exceedingly dark-coloured and dense, and had lost the usual structural appearance of pulmonary tissue.

3. Portions of lungs obtained from the body of a pen-blade grinder. The external surface was thickly studded with small black spots, about the size of currants. Similar bodies were also discovered in the internal structure of the lungs, and here and there they had aggregated so as to form large masses, which were very dense, and retained the impression of the knife when cut into. In the superior lobe, on the left lung there was a large hardened mass, about the size of a hen's egg, which exhibited, when incised, appearances similar to those already noticed. The branches of the vessels, arteries, veins, and bronchi, were considerably dilated, and the lining membrane of the latter much injected. There was slight emphysema at the edges of the lungs, and at the posterior surface of the inferior lobe. The bronchial glands were enlarged, and of a black color, and several of them contained calcareous matter.

4. A drawing, kindly executed by Dr. Branson, of the section of the lung of a fork-grinder, in which a hardened mass about the size of a pigeon's egg, being laid open, presented the appearance of pulmonary apoplexy.

### STONE MASON'S LUNGS.

Dr. Favell also exhibited several sections of the lungs of a stone mason, in which small dark-coloured bodies, similar to those observed in the preceding case, were perceived both on the surface and within the structure of the lung. Several hard masses existed in different portions of the lung, especially in the superior lobe on the right side, which afforded an unusual degree of resistance to the knife, and very closely resembled, both in appearance and density, one of the specimens of grinders' lung. The edges of the lungs were emphysematous. A large cavity, with indurated edges, existed in the superior lobe of the left lung. The bronchial glands were greatly enlarged, indurated, and of a dark colour. Some of the branches of the bronchi were dilated, and the lining membrane injected.—*Prov. Med. Jour.*, *Nov.* 1843.

